# Androgen Deficiency and Phosphodiesterase Type 5 Expression Changes in Aging Male: Therapeutic Implications

**DOI:** 10.3389/fendo.2019.00225

**Published:** 2019-04-11

**Authors:** Antonio Aversa, Ylenia Duca, Rosita Angela Condorelli, Aldo Eugenio Calogero, Sandro La Vignera

**Affiliations:** ^1^Department of Experimental and Clinical Medicine, University “Magna Graecia” of Catanzaro, Catanzaro, Italy; ^2^Department of Clinical and Experimental Medicine, University of Catania, Catania, Italy

**Keywords:** aging, hypogonadism, erectile dysfunction, sexual desire, pde5 expression, pde5 inhibitors, testosterone replacement therapy, elderly

## Abstract

The age-related decline of serum T occurs in ~20–30% of adult men and it is today defined as late-onset hypogonadism (LOH). In the elderly, such decline becomes more prevalent (up to 60%) and shows-up with erectile dysfunction (ED) and hypoactive sexual desire. A large body of experimental evidences have shown that the combination of T replacement therapy (TRT) and phosphodiesterase type 5 inhibitors (PDE5i) is, usually, effective in restoring erectile function in patients with LOH and ED who have not responded to monotherapy for sexual disturbances. In fact, PDE5is potentiate the action of nitric oxide (NO) produced by endothelial cells, resulting in a vasodilator effect, while T facilitates PDE5i effects by increasing the expression of PDE5 in corpora cavernosa. Meta-analytic data have recognized to PDE5i a protective role on the cardiovascular health in patients with decreased left ventricular ejection fraction. In addition, several studies have shown pleiotropic beneficial effects of these drugs throughout the body (i.e., on bones, urogenital tract and cerebral, metabolic, and cardiovascular levels). TRT itself is able to decrease endothelial dysfunction, oxidative stress and inflammation, thus lowering the cardiovascular risk. Furthermore, untreated hypogonadism could be the cause of PDE5i ineffectiveness especially in the elderly. For these reasons, aging men complaining ED who have LOH should undergo TRT before or at the moment when PDE5i treatment is started.

## Introduction

Male hypogonadism is generally characterized by abnormally low serum T (T) levels. Cross-sectional studies have found that 20–64% of old men with diabetes have hypogonadism, with higher prevalence rates found in the elderly. Typical symptoms include sexual dysfunctions, changes in mood, decreased bone mineral density, increased body fat and decreased muscle mass and strength (1). By restoring serum T levels to the normal range using T replacement therapy (TRT), many of these symptoms can be relieved.

A number of other common conditions can also be associated with decreased T production in the elderly. These include metabolic syndrome (MetS), atherosclerosis, myocardial infarction, and chronic heart failure (2). Several studies have shown an increased cardiovascular disease (CVD) risk of up to 4-fold in men with either MetS or type 2 diabetes (3–5). Studies have also shown that low T levels in men can predict the development of insulin resistance, the physio-pathological basis of MetS, and a possible progression to type 2 diabetes (6, 7). Men are twice as likely as women to develop CVD as well as diabetes. This might be ascribe to differences in endogenous sex hormone levels. Indeed, patients with type 2 diabetes mellitus have lower androgen levels and poorer glucose tolerance than non-diabetics (8–10). Thus, low serum T levels are associated with an increase in many of the known cardiovascular risk factors (11) listed in this Table 1.

**Table 1:** Biochemical and metabolic effects of T (T) deficiency and their reversal after T replacement therapy (TRT).

**Low T**		**TRT**	
HDL cholesterol	↓	↓	(Smaller ↓ observed in older men)
Total cholesterol	↑	↓	Total cholesterol
LDL cholesterol	↑	↓	LDL cholesterol
Triglycerides	↑	↓	Apoprotein B
		↓	Lipoprotein a
Hypertension	↑	↓	Diastolic BP by 4–5 mmHg
Fibrinogen	↑	↓	Fibrinogen
PAI-1	↑	↓	PAI-1
Visceral obesity	↑	↓	Visceral obesity
Fasting glucose	↑	↑	Insulin sensitivity
Fasting insulin	↑	↓	Insulin resistance

Adequate T concentrations are also crucial for a proper endothelial function, for the expression of penile PDE5 isoenzyme (12) as well as for the adequate production of hydrogen sulphyde (H_2_S). Thus, long-term TRT would be expected to decrease cardiovascular morbidity and mortality but is not recommended in the frail elderly (13). Men with ED and low T levels are potential candidates to benefit from a combination therapy if response to monotherapy is not sufficient. A combined treatment may result in endothelial rejuvenation by potential remodeling of vascular wall (14). However, since the potential high benefits from these therapies in specific elderly population have not yet been proven, the purpose of this article is to review basic and translational experimental evidences that support a possible role of T in the regulation of PDE5 expression in the urogenital tract and to evaluate its use, alone or in combination for the treatment of patients with LOH and sexual dysfunctions.

## Role of Cyclic Nucleotides and PDEs in T Production and Penile Erection

In Leydig cells (LCs), the production of T is regulated by the cyclic adenosine monophosphate (cAMP) signaling pathway. Luteinizing hormone (LH) binds to its receptors coupled to the G-protein that regulates adenylyl cyclase (ADCYs). This event leads to an increase in the intracellular cAMP levels with subsequent activation of protein kinase A (PKA) that promotes steroidogenesis (15).

The cyclic guanosine monophosphate (cGMP) signaling pathway is also active in LCs and, together with the cAMP signaling pathway, modulates steroidogenesis in LC (16, 17). In these cells, nitric oxide (NO), generated by NO synthases endothelial (eNOS) and/or inducible NO synthase (iNOS), stimulates the production of cGMP. The cGMP, in turn, activates the protein kinase G (PKG) that phosphorylates the acute regulatory steroidogenic protein (StAR), thus promoting steroidogenesis (17, 18).

An inverse relationship between NO production and T secretion has been shown (19). Subsequently, a biphasic relationship was described. Valenti et al. reported that higher concentrations of NO donors decrease T production whereas lower concentrations increase its levels (20). This occurs because at lower concentrations NO activates cGMP-dependent pathway leading to the activation of PKG-1 and consequently the phosphorylation of StAR protein that promotes steroidogenesis (17). Conversely, at higher concentrations, NO directly inhibits the activities of steroidogenic enzymes in LCs (19, 21).

An interaction between the nitrergic and purinergic systems seems to exist in LCs. In fact, recently, it has been shown that basal NO production in LCs changes the adenosine triphosphate (ATP)-evoked currents and that extra NO modulates the current through a mechanism involving the NO/cGMP signaling pathway (22).

The spatiotemporal dynamics of cAMP and cGMP pathways depends upon PDE activity, which by breaking phosphodiesteric bonds terminate cyclic nucleotides signaling (23). In mammals, 11 PDE families exist. These include: PDE4, PDE7, and PDE8 are highly specific for cAMP; PDE5, PDE6, and PDE9 are highly selective for cGMP; while PDE1, PDE2, PDE3, and PDE10 act on both molecules (23).

PDE5A, a cGMP-specific PDE, is expressed in LCs (24) where it seems to modulate cGMP/PKG-stimulated androgen production, as shown by the raise in cGMP and androgen levels after treatment with a selective PDE5i (17). In LCs, T production is also suppressed by the activity of PDE8A, an enzyme that specifically hydrolyzes cAMP. In fact, LCs from PDE8A-null mice secrete about 4-fold more T compared to those of wild-type mice and are more responsive to LH stimulation (25). These data indicate that both cAMP and cGMP are involved in T production, and that PDEs contribute to the regulation of androgen synthesis in LCs and could be target of pharmacological manipulation.

**Figure 1:**
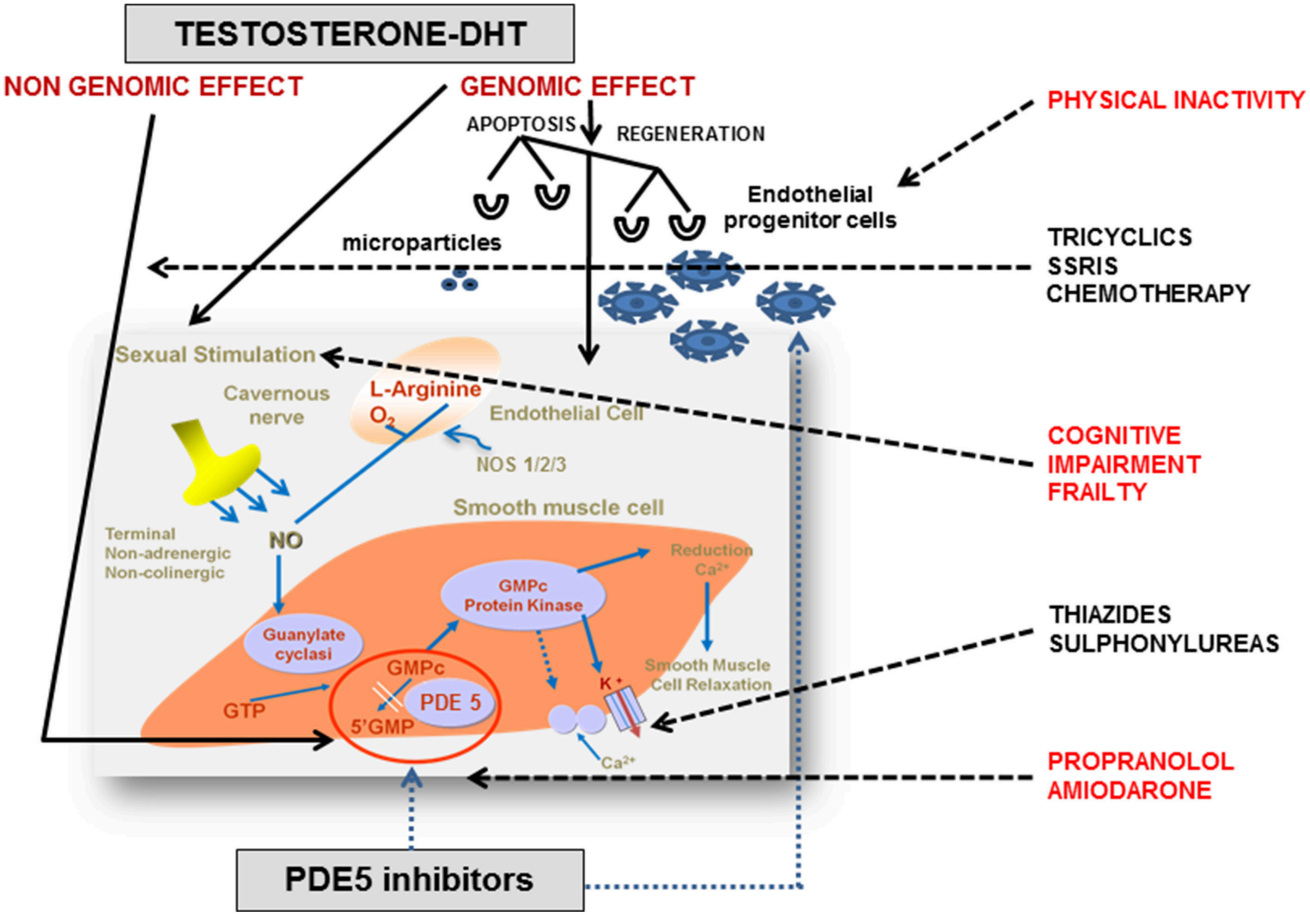
Endocrine and pharmacological regulation of penile erection. In corpora cavernosa the effects of testosterone (T) are primarily mediated by its conversion into 5α-dihydrotestosterone (DHT) and its binding to the androgen receptors (ARs), localized within vascular endothelium and smooth muscle cells. The AR is a ligand-activated transcription factor acting on the genome. The genomic action of AR is modulated by a large variety of co-regulators, that fine-tune target gene expression by enhancing or restraining transcription. However, steroids, including androgens, have also membrane receptors responsible for non-genomic actions. The non-genomic T pathway involves the rapid induction of conventional second messenger signal transduction cascades (i.e., activation of protein kinase A, protein kinase C, and MAPK), leading to diverse cellular effects including smooth muscle relaxation, neuromuscular and junctional signal transmission, and neuronal plasticity. Nitric oxide (NO) is released from nitrergic nerve endings and from the endothelium in response to acetylcholine and to the shear stress due to increased blood flow. Subsequently, NO penetrates into smooth muscle cells and binds to soluble guanylate cyclases, which catalyze the conversion of guanosine triphosphate to cyclic guanosine monophosphate (cGMP). cGMP activates protein kinase G which phosphorylates and activates proteins that reduce the intracellular Ca^2+^ concentration or the sensitivity to Ca^2+^, decreasing muscular tone. Phosphodiesterase type 5 (PDE5) is the predominant enzyme responsible for cGMP hydrolysis in vascular and trabecular smooth muscle. T regulates PDE5 expression and mediates clinical response to PDE5 inhibitors through a putative androgen response element (ARE) in human PDE5A1 promoter. Many interfering factors in the elderly (i.e., frailty, physical inactivity, and polypharmacy) may directly or indirectly block at multiple site the physiological pathways. Adapted from Aversa et al. (28). NOS, nitric oxide synthase; GMP, guanosine monophosphate; GTP, guanosine triphosphate; SSRI, selective serotonin reuptake inhibitors.

cGMP and cAMP are also fundamental in the regulation of the vascular processes that lead to erection. Endothelial cells produce NO, which in turn activates soluble guanylyl cyclase (sGC). The subsequent accumulation of cGMP induces the relaxation of smooth muscle in corpora cavernosa (26). cAMP contributes to erection physiology through the cyclooxygenase-2 (COX-2) pathway. COX-2 and prostacyclin synthase (PTGIS) catalyze the synthesis of prostaglandin E which, by binding to specific receptors on smooth muscle, activates cAMP-dependent pathways that lead to muscular relaxation (27). The main penile regulatory biochemical machinery is summarized in Figure 1.

## Age-Related Changes in T Production and PDE Expression in the Urogenital Tract

It has been shown that serum T levels decrease ~by 1% per year in men from their 30th (29). This is in part due to a progressive age-related decline in T production by LCs. The main causes of this decrease include: a diminished response of cAMP to gonadotropins; a lower LHR expression in LCs; a decline in StAR transcription with consequent impairment of cholesterol intracellular availability; and a decreased activity of some steroidogenic enzymes (30, 31). Another mechanism is the GnRH signaling attenuation in aging men, due to decreased GnRH gene expression and altered pulsatility and amplitude of GnRH pulse (32).

LC of aged rats have lower cAMP concentration whereas NO–cGMP signaling is increased (33). Sokanovic et al. have shown a progressive increase in endogenous NO production in aged rats that contributes to the decrease in T production with a mechanism independent from the cGMP pathway (33). The increase in cGMP alone improves T content in LCs of both adult and aged rats, while an increase in NO levels enhances cGMP and inhibits cAMP production, with consequent T production decrease, but only in LCs from aged rats (33). These findings show that cGMP stimulates and NO inhibits steroidogenesis in aged rats.

Aged animals not only have significantly lower T concentration than adults but they also loose the normal T secretion rhythmicity (31). In LCs of aged rats, the alteration also occurs in the transcription rhythmicity of genes involved in both cAMP and cGMP pathways (34). Aging has been suggested to strengthen the negative cross-talk between the two signaling pathways through changes in the expression of PDEs with dual activity, but these data have to be confirmed (34).

Furthermore, PDE5 gene is over-expressed in LCs of aged rats and its increased activity has been associated with lower T production. Data are less clear in endothelial cells of corpora cavernosa. To our knowledge, data on age-related PDE5 gene expression changes in the corpora cavernosa are not available. Indirect information comes from studies that investigated the effects of hypogonadism and TRT on PDE5 gene expression. Lower T levels are one of the features of aging but it is not always present in elderly men and, furthermore, other mechanisms, independent from T, could be involved in the regulation of the expression of PDE genes in the elderly. Up-regulation of PDE5 gene is believed to be one of the mechanisms underlying androgen therapeutic effects in the treatment of ED (35–37). This belief derives from the finding of a putative presence of the androgen response element (ARE) in the human PDE5A1 gene promoter (38). However, more recently, the same authors have criticized the results of their previous studies. In fact, the up-regulation of the PDE5 gene by T would create a paradox in which a positive regulator of erectile function (androgen) would increase the level of a negative regulator (PDE5), potentially leading to worsening of ED and to a more difficult clinical management (39). Moreover, if so, in the corpora cavernosa would occur the exact opposite of what happens in LCs, where aging-related T decrease is associated to an increased expression of PDE5 (34). Finally, two studies that have looked for androgen-responsive genes in the whole human genome and they found respectively 524 and 1,532 potential AR-binding sites, but PDE5A gene was not among them (40, 41). Therefore, further studies are needed to clarify the relationship between androgens and PDE5 gene expression in the corpus cavernosum, but it is well-established that TRT improves the effect of PDE5i treatment in patients with hypogonadism (see Synergic Effect of T Plus PDE5is in the Treatment of Erectile Dysfunction in Patients with LOH).

cGMP pathway also plays a fundamental role in bladder, prostate, seminal vesicle and epididymis physiology. In these organs, the cGMP-signaling pathway regulates muscle contractions and peristalsis, cell proliferation, and secretory activity (42). The urogenital organ with the most active cGMP signaling pathway seems to be the bladder, where NO–cGMP pathway regulates the micturition reflex and the phasic contractile activity (43, 44). A study revealed that bladder shows high expression of PDE5 and that the amount of this protein is significantly lower in aged bladder than in younger ones, probably due to the age-related decrease in muscular content (42). These findings remark the pivotal role played by cGMP pathway in the physiology of the bladder, which could therefore represent a favorable target for PDE5i pharmacological action (42).

With aging, prostate progressively develops benign prostatic hyperplasia (BPH), that is present in up to 90% of men over 80 years of age (45). BPH is characterized by enlargement and alteration of stromal compartment, focal proliferation of smooth muscle cells, epithelial basal cell hyperplasia, and nodular arrangement of the transition zone of the gland (46). Development of BPH is multifactorial; one of the pathophysiological mechanisms involves the increase in estrogen/androgen ratio in prostatic stromal tissue, due to the lower T production and conversion to DHT. Other factors are the interaction between growth factors (IGF, FGF, TGF) and steroid hormones and chronic prostate inflammation (47). A study has shown that prostate is an organ with poor expression of all enzymes implicated in cGMP signaling pathways, including PDE5; but PKG1 expression shows an age-related increase in rat prostate cells (42). In the same study, Authors, considering the pronounced androgen dependency of prostate, investigated the expression of cGMP pathway proteins in this tissue in conditions of androgen deprivation. Interestingly, they showed a further upregulation of PKG1 and a less pronounced increase in PDE5 expression (42), similarly to what Baburski et al. showed in LCs of aged rats (34). At the prostate level, the cGMP pathway could be implicated in the relaxing activity and in the regulation of proliferation and differentiation of smooth muscle cells. In fact, PDE5is showed the ability to lower the proliferation of prostate stromal cells and fibroblast-to-myofibroblast trans-differentiation (48). Authors, therefore, speculated that PKG1 could be directly implicated in cellular proliferation processes: decreased androgen levels could increase prostatic PKG1 expression and, in turn, promote cell proliferation (42). Another study has shown an up-regulation of PDE5 in both rat and human BPH, which was immunolocalized in prostate fibromuscular stroma. Since BPH was obtained in experimental models by T administration, the authors speculated that the increased PDE5 expression could be due to the increase in T levels (49), partly contradicting the results of Müller et al. that showed an increase in PDE5 expression following T deprivation. BPH is an androgen-dependent disease. In fact, androgen ablation (by administration of GnRH agonists, androgen receptor antagonists or DHT inhibitors) is an effective strategy in decreasing prostate volume. We also speculate that these beneficial effects may be mediated by a decreased expression of PDE5, which is androgen-dependent in the rat bladder, and therefore by an enhancement of NO-induced relaxation during the filling phase. This latter aspect may account for the beneficial effect of daily PDE5i use on detrusor overactivity (50). Nevertheless, further investigations are needed to clarify the mechanism that regulate PDE5 expression in prostate cells.

## Late-Onset Hypogonadism and Erectile Dysfunction

The age-related T decline, known in the past as male menopause or andropause, is today defined as late-onset hypogonadism (LOH) (29, 32). Hypogonadism is diagnosed when at least two T measurements, obtained from morning blood samples, are low in the presence of signs and symptoms of androgen deficiency (51). The most specific symptoms associated with LOH are the sexual ones: decreased frequency of morning erection, decreased frequency of sexual thoughts, and ED (52).

ED in hypogonadal patients is strictly related to systemic endothelial dysfunction. In fact, T is able to promote angiogenesis and endothelial cell proliferation through a mechanism mediated by the androgen receptor (AR)/vascular endothelial growth factor (VEGF) pathway (53). Endothelial microparticles (EMPs) are fragments of the plasma membrane released from the injured vessels and are considered a marker of endothelial dysfunction. Their concentration increases in patients with LOH and ED (54). Endothelial progenitor cells (EPCs) are a group of cells, similar to the embryonic angioblasts, that can originate from the mesoderm or from transdifferentiated monocyte/macrophages. EPCs could have different possible phenotypes and are implicated in the vasculogenic reparative process and the consequent re-endothelization after vascular injuries (54). Some EPC populations are decreased in hypogonadal patients (55), while other EPCs subtypes are present in higher concentration in patients with hypogonadism and ED, compared to eugonadal patients with ED (56). This last subpopulation of EPCs (i.e., EPCs CD45neg/CD34pos/CD144pos) could be considered a marker of vascular damage, in response to which they are produced in greater quantities in the attempt to repair the injured endothelium. In fact, they have been found in higher concentrations in the blood of patients with coronary artery disease, and their levels increase as ED worsens (54, 57). Furthermore, it has been shown that a greater endothelial damage is related to a worse pharmacological response to PDE5i (58).

The oxidative stress is another mechanism by which hypogonadism affects endothelial function. In fact, it has been shown that hypogonadism increases oxidative stress and decreases NO bioavailability (59–61). Nicotinamide adenine dinucleotide phosphate (NADPH) oxidase is an enzymatic complex that catalyzes the production of reactive oxygen species (ROS) (62). In castrated rats some NADPH oxidase subunits are over-expressed and this up-regulation leads to increased ROS production in the corpora cavernosa (63). In this model, TRT lowers the expression of NADPH oxidase and, consequently, ROS production, increases NO bioavailability and improves erectile function (63). In the same study, the Authors showed that hypogonadism decreased the expression of COX-2 and PTGIS, leading to a decreased penile cAMP levels. TRT also restores COX-2 and PTGIS expression, and increases cAMP concentration (63). Therefore, the decrease of ROS production and the activation of COX-2/PTGIS/cAMP signaling pathway with consequent increase in cAMP production might represent two mechanisms through which TRT may restore erectile function in hypogonadal patients.

Hypogonadism is associated with a low-grade inflammation that may be involved in the pathogenesis of androgen deficiency symptoms in aging men. A correlation between C-reactive protein (CRP) and aging male symptom (AMS) score has been reported, and a reduction in CRP levels and AMS scores was shown following TRT (64). The concentrations of IL-6, NF-Kb mRNA, and asymmetric dimethylarginine (ADMA), an endogenous NO synthase inhibitor that increases in response to inflammation, have been shown to be higher in castrated rats compared to controls (65). T administration to castrated rat decreases these markers of inflammation suggesting that T deficiency could increase oxidative stress and endothelial dysfunction by stimulating inflammation (65).

The endothelial dysfunction in hypogonadism is a systemic event, not exclusively confined to the penile district. Several epidemiological and observational studies have shown that low T levels are associated with cardiovascular diseases as atherosclerosis, coronary artery disease, and coronary events (66). A meta-analysis of 70 studies found significantly lower T levels in patients with cardiovascular diseases than controls (67). Finally, ED itself, one of the main symptoms of hypogonadism, is an independent risk factor for cardiovascular disease and it predicts the presence and the extent of subclinical atherosclerosis (68). In the aging male with LOH, the endothelial damage related to hypogonadism is added to the age-related endothelial dysfunction due to an imbalance between oxidative stress and antioxidant status, which predisposes elderly patients to cardiovascular events (69).

It is noteworthy that systemic endothelial dysfunction and atherosclerosis can also affect the microcirculation of the testis and cause a LC dysregulation with consequent lower T production (70). Therefore, a vicious circle could be established: in the elderly, LOH worsen age-related endothelial dysfunction that leads to a further T production decrease by affecting testicular microcirculation.

## Synergic Effects of T Plus PDE5is in the Treatment of Erectile Dysfunction in Patients With LOH

PDE5is are the first choice drugs for the medical treatment of ED (71, 72). PDE5is inhibit the effect of PDE5, which terminates cGMP's effects breaking down its phosphodiester bond. The consequent intracellular accumulation of cGMP activates cGMP-dependent protein kinase which phosphorylates specific proteins implicated in a number of physiological responses, such as smooth muscle relaxation, platelet aggregation, and cardiac functions (73). In corpora cavernosa, NO is released from nitrergic nerve endings, from the endothelium in response to acetylcholine released by parasympathetic endothelial nerve endings, and by the shear stress due to increased blood flow in the sinusoids. NO penetrates into smooth muscle cells and binds to sGC, which catalyze the conversion of guanosine triphosphate to cGMP. cGMP activates PKG which phosphorylates and activates proteins that reduce the intracellular Ca^2+^ concentration or the sensitivity to Ca^2+^, decreasing, consequently, the muscular tone (73). The final result is vasodilatation and an increased blood flow into the cavernosal sinusoids which leads to erection. PDE5is potentiate this effect by increasing the level of intracellular cGMP when the NO-signaling pathway is activated.

PDE5is currently available (sildenafil, vardenafil, tadalafil, avanafil, mirodenafil, udenafil, and lodenafil) show the same pharmacodynamics, but they differ from each other for the pharmacokinetic properties. Avanafil and vardenafil have the quickest onset of action, whereas tadalafil shows the longest half-life (up to 36 h) (72). This favorable pharmacokinetic feature allowed to approve tadalafil for daily use, while the other PDE5is are usually administrated on-demand.

About 30% of patients are poor responders to PDE5is (74). One of the causes that can impair the response to PDE5i is indeed hypogonadism (75). In the corpora cavernosa of experimental models, the androgen deprivation causes smooth muscle cell apoptosis and adipose tissue deposition with consequent fibrosis; decreased expression of eNOS and neuronal nitric oxide synthases; decreased arterial inflow and increased venous outflow; enhanced response to vasoconstrictor mediators such as α-adrenergic agents; and decreased NO-mediated smooth muscle relaxation after sexual stimulation (75).

Another modification ascribed to hypogonadism is a decreased PDE5 expression (see Figure 1). Some studies did not confirm the presence of an ARE in the human PDE5A gene promoter initially described (39), but several studies anyway reported a down regulation of PDE5 expression in the corpora cavernosa, prostate and bladder in animal models after surgical or pharmacological castration. In all these studies, PDE5 expression was restored by T administration (12, 76–78). It has been reported that castration lowers the content of smooth muscle cells in the corpus cavernosum and prostate, which are replaced by non-muscular cells such as adipocytes (79, 80). PDE5 is expressed in smooth muscle cells of corpora cavernosa, resulting in a lower amount of substrate on which PDE5is may act, making less effective the action of these drugs. Thus, TRT could facilitate the pharmacological effects of PDE5is, restoring the structure of the corpora cavernosa and increasing their content in smooth muscle cells and, consequently, the expression of PDE5 (39). Furthermore, this would explain the temporal interval (up to 6 months) necessary for T to improve erectile function, as the structural modifications of the corpora cavernosa induced by TRT require time to be completed (81).

TRT has also been proven effective in lowering circulating EPC and EMP concentrations and in restoring NO levels in corpora cavernosa (82–84). This suggests that TRT improves erectile function by decreasing the degree of endothelial damage. A similar effect seems to be produced by physical activity. Indeed, a recent study showed that in patients with LOH a protocol of 150 min per week of moderate-intensity aerobic exercise in association with tadalafil 5 mg daily for 90 days improves erectile function even if total blood T levels are below normal. The improvement of erectile function was shown by an increased international index of erectile function 5 (IIEF5) score and the main vascular arterial parameters (acceleration time and peak systolic velocity) evaluated by penile Doppler ultrasound (85).

Increased efficacy of combined T and PDE5i administration compared to PDE5 monotherapy in hypogonadal patients has been shown by several studies (86–93). Furthermore, the administration of T undecanoate plus once-daily tadalafil 5 mg is more effective in restoring erectile function than the combination of T undecanoate plus on-demand tadalafil 10–20 mg. Over 30 weeks of treatment, patients treated with T undecanoate plus once-daily tadalafil 5 mg showed higher IIEF5 and AMS scores and, after 6 weeks from treatment discontinuation, a higher percentage of patients had a maintenance of their subjective erectile function improvement (94).

**Figure 2:**
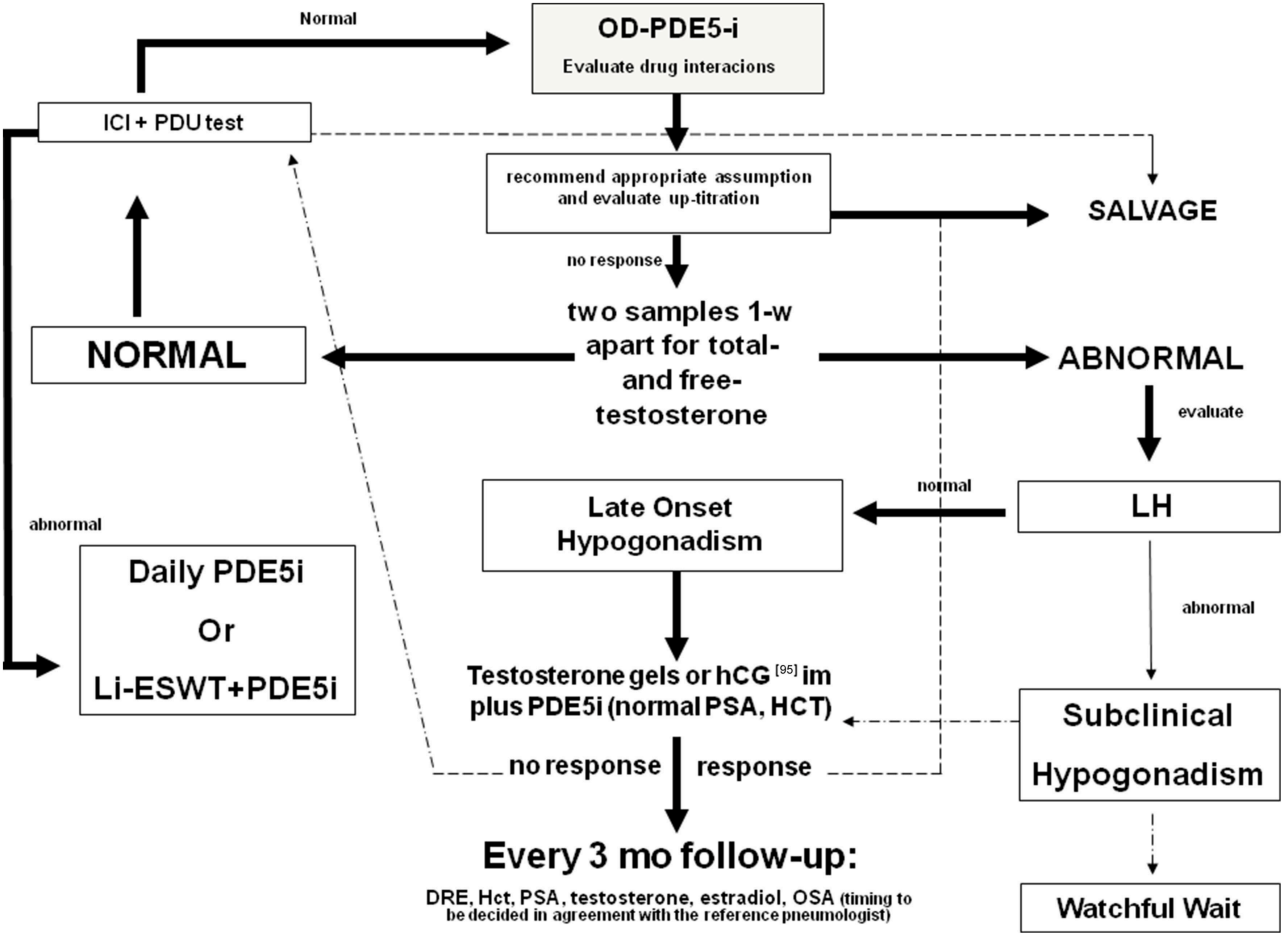
Diagnostic flow-chart for the elderly patient with sexual dysfunction not responding to oral PDE5i. Testosterone (T) deficiency could be a cause of non-response to treatment with PDE5i. Therefore, in patients with hypogonadism any treatment for erectile dysfunction (ED) should be initiated with T, whereas PDE5is could be co-administered for immediate relief of poor erection complaints. In the elderly, T levels must be preferably restored by the administration of T in gel formulation. Patients with normal gonadotropin levels could also benefit by hCG administration. Follow-up must be performed every 3 months evaluating PSA, blood count, and androgen levels. A digital rectal examination and a pneumological evaluation could also be indicated. In patients with normal blood T levels, the prescription of PDE5is alone on demand is always recommended. If the patient does not respond to this treatment, the second step is to evaluate any drug interaction, the correct PDE5i intake, and the eventual drug up-titration. Penile pharmaco-testing with alprostadil (ICI) followed by penile dynamic Duplex ultrasound (PDU) may retrieve important data regarding vascular health and stratification of cardiovascular risk as well as possible therapeutic approaches (96). These include daily PDE5is, intra-cavernous or intra-urethral alprostadil administration, and low-intensity extracorporeal shockwave therapy [adapted from Aversa et al. (75)]. OD, on demand; DRE, digital rectal examination; PSA, prostate specific antigen; Li-ESWT, low-intensity extracorporeal shockwave therapy; OSA, obstructive sleep apnea.

The synergic effect of T plus PDE5i seems to be evident also when patients begin TRT at first (Figure 2). Yassin et al. showed that just under 50% of hypogonadal patients with ED fail to respond to T undecanoate treatment alone within 3 months. Almost all of these patients respond well to the addition of 20 mg vardenafil on demand (97). In another study, hypogonadal patients received 1% T gel and 100 mg sildenafil was added to those who did not obtain an improvement in erectile function after 3 months of therapy. All these patients responded well to the combination therapy (98). A bias of these two studies may be the duration of TRT before the addition of PDE5is, because in some patients (those with more severe or long-standing hypogonadism), the therapeutic efficacy of TRT may become evident after more than 12 weeks (81).

Recent evidence showed that PDE5is, until recently considered a symptomatic therapy for ED, can partly exert an effect on the pathophysiological mechanisms that lead to LOH. Sokanovic et al. treated aged rats with oral sildenafil, a specific inhibitor of PDE5, and, after 3 and 6 months of therapy, they found an improvement in steroidogenesis. The Authors showed an increase in cGMP/NO ratio, a decreased serum nitrite levels, and an increased cAMP content of LCs (33). They also analyzed PDE gene expression in LCs from sildenafil-treated animals and controls. Aging produced alterations in the pattern of PDE gene expression and treatment with PDE5is was able to reverse these alterations, contributing to the normalization of cAMP levels. Finally, sildenafil treatment increased the transcription of key genes for steroidogenesis (CYP11a, CYP17a1, HSD3b, HSD17b4, and StAR) probably contributing to the increase in androgen levels found in aged rats treated with sildenafil (33). In a recent study, the same group reported that long-term PDE5 inhibition slows-down the regressive changes that take place in testes during aging (99). Similar results have also been reported in humans. For example, Spitzer et al. found that sildenafil administration in patients with ED and low serum T levels leads to a significant increase in total and free T and a decreased serum LH concentration, suggesting a direct effect of PDE5is at the testicular level (100).

Overall, these data indicate that T and PDE5i act synergistically to improve erectile function in patients with LOH. Chronic androgen deprivation leads to anatomical and histological alterations of the corpora cavernosa that make the pharmacological action of PDE5is sometimes ineffective (see diagnostic algorithm in Figure 2). For this reason, it is more appropriate to normalize T levels before starting PDE5i administration, taking into account that the structural improvement of the corpora cavernosa and, consequently, of the erection can take up to 6 months to occur (81). In parallel, PDE5is have been shown to improve T levels in patients with LOH and, in animal models, to prevent or slow-down the regressive changes, partially responsible for the onset of LOH, that occur in the testis during aging. Therefore, PDE5i therapy could prevent aging-related testicular alterations (and then LOH) and in clinical hypogonadism may be effective in normalizing androgen levels in association with TRT.

## Conclusions

Aging leads to a progressive decrease in androgen production that, in turn, leads to the development of LOH, defined by significant low T serum levels (in the lowest quartile) in the presence of signs and symptoms of hypogonadism (51). LOH could be due to both testicular and hypothalamic-pituitary dysfunction (32), and ED is one of its main symptoms. ED in LOH is linked to increased oxidative stress, subclinical inflammation, and subsequent endothelial dysfunction (101). In elderly men, it has been shown that LOH is also linked to lower cAMP pool and to an alteration of the cGMP signaling pathway.

PDE5 gene lower expression is associated to aging and hypogonadism at the corpus cavernosum level. TRT is able to restore the expression of PDE5 gene and this effect is initially attributed to a direct regulation of the gene expression by T (38). Subsequently, this hypothesis was not confirmed, and the authors hypothesized that the lower expression of PDE5 in hypogonadism was due to the decreased smooth muscle cell content in corpora cavernosa. Therefore, T could able to increase PDE5 content by reversing these anatomical changes (39). Anyway, the increased PDE5 gene expression explains the reason for the possible failure of PDE5i administration in hypogonadal patients with ED.

The timing of treatment with T and PDE5is in patients with hypogonadism and ED is a matter of debate (102). The initial approach to patients with ED encompasses the use of PDE5is (72) (Figure 2). However, as we have seen, hypogonadism is, especially in aging men, a common cause of ED and a reason for a lack of response to PDE5is (71). Hence, patients with ED should be tested for androgen deficiency before treatment with PDE5i is given (102), because TRT it is effective in about half of the patients with ED (84). The addition of PDE5is should be reserved to those patients in whom ED persists despite the eugonadal state restoration. However, the time-course of T effects requires long-term administration to become detectable (81).

PDE5is showed the ability to enhance steroidogenesis at the testicular level, to reverse the age-related alterations of PDE genes expression (33, 100), and to slow-down age-related regressive alteration of the testis (33). Furthermore, PDE5is have pleiotropic actions throughout the body that could counteract the age-related physiopathological alterations that affect the urological tract and male accessory sexual glands (48, 103), bone (104), fat tissue (105), brain (106), and heart (107).

Before starting any treatment, elderly men should be accurately investigated for the presence of major contraindications to the use of TRT and/or PDE5is even in the presence of hypogonadism. Once this work-up is completed, treatment(s) should be wisely offered to improve their sexual function whenever cardiovascular efficiency is proven.

From a clinical-translational point of view, the information provided in this review would suggest careful consideration of the systemic implications of hypogonadism in the elderly and the benefits of treatment since there is disagreement on the threshold value for its safe prescription. In summary, we suggest a total T value <8 nmol/L along with uncompensated LH levels and relevant clinical symptoms i.e., sexual symptoms, sarcopenia, anemia, osteoporosis (12–14, 36, 37, 50, 75, 81).

Several studies have shown an inverse relationship between indicators of obesity (body mass index, waist circumference, a reliable indicator of visceral obesity), DM2/metabolic syndrome and T levels over all age groups. Hence, erectile dysfunction may be considered a predictor of severe peripheral vascular damage when compared to healthy population and should be regarded as a major health threaten for the older patients (47, 54, 57, 58, 83, 85, 101, 103).

## Author Contributions

AA and SL conceived the idea and revised the manuscript. YD wrote the manuscript. AC and RC performed the literature search, and corrected syntax and typos.

### Conflict of Interest Statement

The authors declare that the research was conducted in the absence of any commercial or financial relationships that could be construed as a potential conflict of interest.

## Abbreviations

ADCY: adenylyl cyclase
ADMA: asymmetric dimethylarginine
AMS: Aging Male Symptom
AR: androgen receptor
ARE: androgen-response element
ATP: adenosine triphosphate
BPH: benign prostatic hyperplasia
cAMP: cyclic adenosine monophosphate
cGMP: cyclic guanosine monophosphate
COX-2: cyclooxygenase-2
CRP: C-reactive protein
CVD: cardiovascular diseases
ED: erectile dysfunction
EMPs: endothelial microparticles
eNOS: endothelial nitric oxide synthases
EPC: endothelial progenitor cells
GnRH: gonadotropin-releasing hormone
H_2_S: hydrogen sulphyde
IIEF5: International Index of Erectile Function 5
iNOS: inducible nitric oxide synthases
LCs: Leydig cells
LH: luteinizing hormone
LHR: luteinizing hormone receptor
MetS: metabolic syndrome
NADPH: nicotinamideadenine dinucleotue phosphate
nNOS: neuronal nitric oxide synthases
NO: nitric oxide
PDE: phosphodiesterase
PDE5i: phosphodiesterase 5 inhibitors
PKA: protein kinase A
PKG: protein kinase G
PTGIS: prostacyclin synthase
ROS: reactive oxygen species
sGC: soluble guanylyl cyclase
StAR protein: steroidogenic acute regulatory protein
T: T
TRT: T replacement therapy
VEGF: vascular endothelial growth factor.
